# CD3^+^CD4^−^CD8^−^ T cells: a new potential therapeutic target in treating autoimmune diseases

**DOI:** 10.3389/fimmu.2025.1683418

**Published:** 2025-09-26

**Authors:** Xin Li, Di Guo, Isabelle Xinyue Zou, Lingyan Zhao, Na Yang, Yang Liu

**Affiliations:** ^1^ College of Basic Medical Sciences, Shanxi University of Chinese Medicine, Jinzhong, China; ^2^ Basic Laboratory of Integrated Traditional Chinese and Western Medicine, Shanxi University of Chinese Medicine, Jinzhong, China; ^3^ Engineering Research Center of Cross Innovation for Chinese Traditional Medicine of Shanxi Province, Jinzhong, China; ^4^ Department of Acute General Medicine, John Radcliffe Hospital, Oxford, United Kingdom

**Keywords:** autoimmune diseases, ontogeny, heterogeneity, traditional Chinese medicine, immunotherapy, CD3^+^CD4^−^CD8^−^ T cells

## Abstract

As a key lymphocyte population in shaping and controlling adaptive immune response, T cells play an important immunomodulatory role in the early stages of autoimmune diseases. Although CD3^+^CD4^−^CD8^−^ T (DNT) cells constitute only a small proportion of peripheral T lymphocytes, they may be closely linked to the occurrence and development of autoimmune diseases. However, the role of DNT cells in autoimmune disease pathogenesis still needs to be elucidated. In this review, we first present the origin, functions, and heterogeneity of DNT cells. We then summarize the role of DNT cells in the pathogenesis of various autoimmune diseases. Subsequently, we clarify the recent advances in the applications of DNT cell-based therapy for autoimmune diseases and outline potential drugs (including active ingredients extracted from Chinese medicinal treatments) and approaches that can target the proliferation and expansion of DNT cells. Lastly, the limitations and challenges of applying DNT-cell-based therapy are analyzed. In conclusion, we present an overview to further the understanding of the role of DNT cells in autoimmune disease pathogenesis and of DNT cells as a potential therapeutic tool for immune disorders.

## 1 Introduction

It is widely accepted that CD4^+^ helper and CD8^+^ cytotoxic T cells form the majority of adaptive immune cells, which shape and control the immune response, and play crucial roles in the development of immune diseases and tumors (1). They are characterized by the positive expression of T-cell receptor (TCR) (alpha and beta chains, αβ) and either CD4 or CD8 (2). The aberrant activation and accumulation of the CD4^+^ and CD8^+^ T cells are the foremost hallmarks of autoimmune diseases (3). However, another small population of T cells, termed CD3^+^CD4^−^CD8^−^ T cells (Double-Negative T cells, DNT), has gained attention for its contribution to the pathophysiology of several autoimmune diseases (4, 5).

DNT cells are T cells which express CD3 but lack both CD4 and CD8 coreceptors (6). They also do not express NK cell markers such as CD56 (7). DNT cells do express TCR αβ or TCR gamma and delta (γδ), which enables them to recognize and respond to pathogens, mounting a functional adaptive immune response (7, 8). In humans, DNT cells make up approximately 1-5% of the total lymphocyte population in the peripheral blood and lymphoid organs (9). DNT cells, like CD4^+^ and CD8^+^ T cells, possess innate and adaptive immune functions, and hence play an important role in the pathogenesis of autoimmune diseases, including in autoimmune lymphoproliferative syndrome (ALPS), systemic lupus erythematosus (SLE), systemic sclerosis, and Sjogren’s syndrome (10–13). Nevertheless, our understanding of the functions and cellular origins of DNT cells remains limited.

Here, DNT cells play diverse roles in autoimmune diseases and immunotherapy, with potential molecular mechanisms. We will also discuss the potential combination of Chinese medicinal materials and DNT Cells in treating autoimmune diseases. This review aims to broaden current understanding of the complex roles of DNT cells.

## 2 Ontogeny and classification of DNT cells

### 2.1 Ontogeny of DNT cells

Progenitor lymphocytes originate in the bone marrow and generally migrate to the thymus to undergo differentiation into mature T cells. Developing T cells progress through multiple stages of differentiation, defined by the expression or lack of the CD4 and CD8 coreceptor molecules, including the Double Negative stage (DN), Double Positive stage (DP), and Single Positive stage (SP) (14). According to the expression of CD25 and CD44, murine DN thymocytes are further divided into four differentiation stages, which are DN1 (CD44^+^CD25^-^), DN2 (CD44^+^CD25^+^), DN3 (CD44^-^CD25^+^), and DN4 (CD44^-^CD25^-^). Human DN thymocytes undergo three distinct DN stages (DN1–DN3), defined by the expression or lack of CD34, CD38 and CD1a (15).

**Figure 1:**
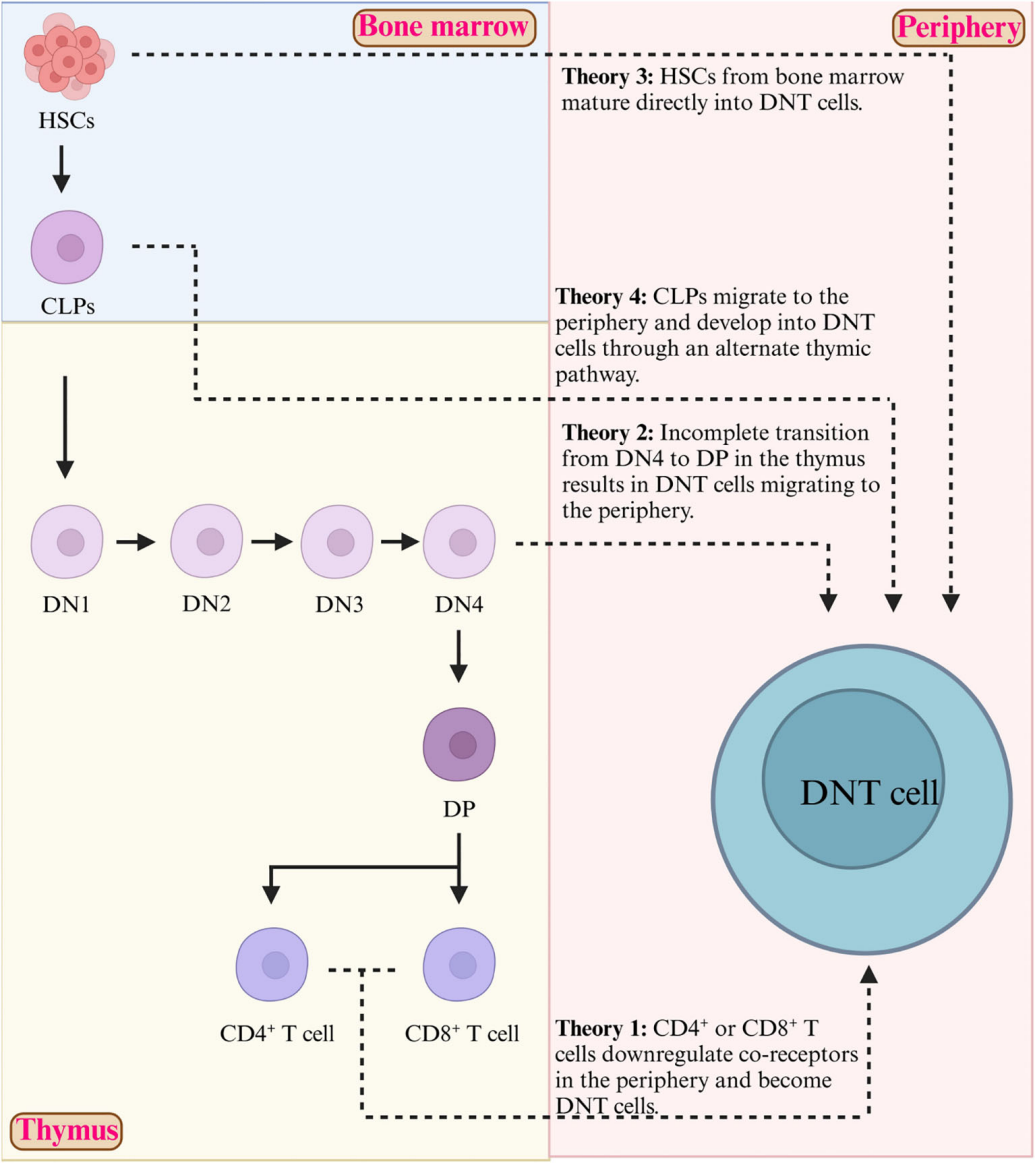
Proposed mechanisms of DNT cell development. Four proposed theories exist on the origin of DNT cells. Theory 1 suggests that CD4^+^ or CD8^+^ T cells in the periphery may downregulate their co-receptors, possibly through demethylation, leading to the formation of DNT cells. Theory 2 proposes that an incomplete transition from DN4 to C stages in the thymus results in DNT cells migrating to the periphery. Theory 3 posits that HSCs directly mature into DNT cells in the periphery. Theory 4 indicates that CLPs migrate to the periphery and develop into DNT cells utilizing an alternate thymic pathway. DN, double negative stage; DP, double positive; HSCs, hematopoietic stem cells; CLPs, common lymphoid progenitors.

There is evidence that early T cells may develop into mature DNT cells in both the thymic and peripheral environments, however, the origin of DNT cells is still poorly understood (16). There are multiple theories regarding this, as shown in **Figure 1**. Peripheral DNT cells may originate from immature DN thymocytes which do not undergo negative selection in the thymus, and hence they escape further thymic development and migrate to peripheral blood. One theory hypothesizes that T cells which express CD4 or CD8 become DNT cells in the periphery by downregulating the expression of the co-receptor (possibly by demethylation of the CD8 or CD4 genes) (17). A second theory is that the DNT cells do not complete differentiation from DN4 to DP T cells, and instead exit the thymus and enter the periphery (18). There is a third theory that hematopoietic stem cells can leave the bone marrow, bypassing the thymus and mature into DNT cells in the periphery without needing to first develop from CD4^+^ or CD8^+^ T cell precursors (19). A fourth theory suggests that common lymphoid progenitor (early T) cells underwent an alternative developmental pathway in the thymus, hence not developing further into CD4^+^ or CD8^+^ T cells (9). A major limitation in understanding DNT cell ontogeny is that most studies use murine models, many of which are transgenic. Therefore, the application of this data to human immunology should be undertaken with caution.

### 2.2 Classification and function of DNT cells

**Figure 2:**
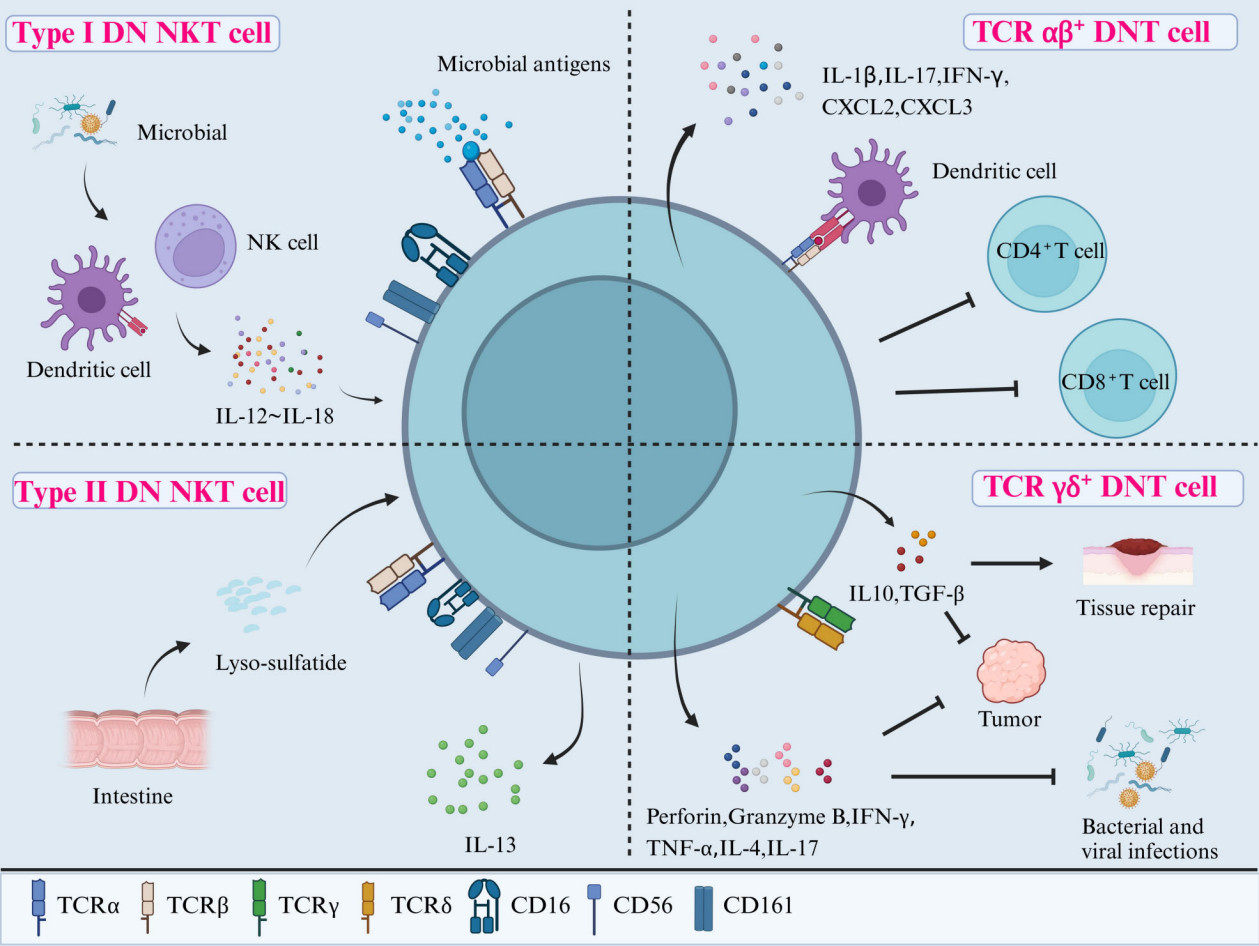
Classification and functions of DNT cells. DNT cells can be divided into three subpopulations according to their cell surface receptors: DN NKT cells (including Type I and II), TCR αβ+ DNT cells, and TCR γδ+ DNT cells. Type I DN NKT cells may protect the host from infections after being activated through the direct recognition of microbial antigens via TCR or by cytokines (ranging from IL-12 to IL-18) secreted from DCs and NK cells in response to microbial antigens. Type II DN NKT cells interact with tissue-specific antigens, such as lyso-sulfatide, to regulate the immune reactions in the gut and other tissues. TCR αβ^+^ DNT cells have pro-inflammatory roles through producing cytokines such as IL-1β, IL-17, and IFN-γ. They can also exert potent immunosuppressive activity on CD4^+^ and CD8^+^ T cells after activation by APCs. Lastly, TCR γδ^+^ DNT cells play a role in combatting infections and tumors by releasing perforin, granzyme B, IFN-γ, TNF-α, IL-4, and IL-17, as well as promoting tissue repair via IL-10 and TGF-β. NKT, natural killer T cells; IL, interleukin; DCs, dendritic cells; TCR, T cell receptor; IFN-γ, interferon-γ; TNF-α, tumor necrosis factor-α; TGF-β, transforming growth factor-β.

DNT cells are difficult to characterize into different phenotypes because there is a lack of well-defined cell markers, and thus, exclusion criteria are often used instead. As shown in **Figure 2**, DNT cells can be divided into three subpopulations that include DN natural killer T cells (NKT), TCR αβ^+^ DNT cells, and TCR γδ^+^ DNT cells (20). DN NKT cells have a close relationship with autoimmunity and infection. TCR αβ^+^ DNT cells can have both inflammatory and anti-inflammatory functions. TCR γδ^+^ DNT cells have potential roles in cytotoxicity against tumor cells, as well as bacterial and viral infections.

#### 2.2.1 DN NKT cells

DN NKT cells express NK-related surface markers, such as CD16, CD56, and CD161, as well as the TCR (21). They can be further divided into DN NKT type I and II cells. Type I DN NKT cells (also known as invariant natural killer T cells (iNKT) have a highly conserved TCR α chain and therefore are more restricted in their TCR repertoire (22). Type I DN NKT cells are activated by lipid antigens presented by CD1d (a non-classical major histocompatibility complex (MHC) member), for example, human and murine type DN NKT cells recognize marine sponge glycolipid and α-galactosylceramide (α-GalCer) (22). Once activated, they have a cytotoxic action within minutes (23). In contrast, the type II DN NKT cells are not activated by α-GalCer, but do react with a wider range of lipid antigens, including self-lipids. For example, they are activated by sulfatide, a glycolipid in myelin found in the central nervous system, when presented by CD1d, which could play a role in autoimmune demyelinating diseases (24).

Type I DN NKT cells can be activated by antigens through two different mechanisms: a. direct binding of TCR to microbial antigens presented by CD1d, known as direct recognition; and b. expansion mediated by cytokines (IL-12 and IL-18) released by other cells (antigen-presenting cells like dendritic cells, other NK cells, other T cells), known as indirect recognition. The cytokine response in indirect recognition can lower the threshold for TCR-mediated activation of NKT cells, however, there is also evidence to show that IL-12 and IL-18 alone can activate NKT cells without the need for CD1d-mediated antigen binding (25, 26). The type II DN NKT cells, also named sulfatide-reacting NKT cells, also recognize antigens through CD1d-mediated TCR activation. They may display protective or pro-inflammatory functions according to the surrounding types of tissue-specific ligands. For example, activation of type II DN NKT cells in a murine CNS model prevented induction of autoimmune encephalomyelitis, potentially through inducing the anergy of type I DN NKT cell and dendritic cells (27). Conversely, type II DN NKT cells in the gut responded to stimulation by lyso-sulfatide by secreting IL-13 and inducing epithelial cell cytotoxicity, thought to play a role in the pathogenesis of ulcerative colitis (28).

#### 2.2.2 TCR αβ^+^ DNT

TCR αβ^+^ DNT cells are characterized by the expression of polyclonal TCR αβ molecules without NK cell-related markers (such as CD16 and CD56). They also do not express regulatory T (Treg) cell-related markers (such as CTLA-4 and CD25) (7). Furthermore, some inhibitory molecules, including programmed cell death protein (PD-1) and Helios, are highly expressed by TCR αβ^+^ DNT cells. Studies in murine models have shown that PD-1 and Helios are expressed by DNT cells derived from self-reactive CD8^+^ T cells, through loss of CD8 expression after self-antigen recognition (29).

TCR αβ^+^ DNT cells, especially a phenotype derived from self-reactive CD8^+^ T cells, can produce an array of pro-inflammatory mediators including IL-1β, IL-17 and interferon (IFN)-γ (30). On the other hand, TCR αβ^+^ DNT cells are a type of inducible Treg cell and can also exert potent immunosuppressive activity. Activated human TCR αβ^+^ DNT cells can reduce the proliferation and cytokine production of CD4^+^ and CD8^+^ T cells, thought to be through a cell contact-dependent mechanism and TCR binding (31). Murine TCR αβ^+^ DNT cells have also been found to contribute to peripheral tolerance to alloantigens *in vitro* and *in vivo* by killing activated syngeneic CD8^+^ T cells through the Fas-FasL pathway (32). Another function of TCR-αβ^+^ DNT cells is that they make up a subset of the mucosal-associated invariant T-cell family (MAIT), where they can respond to mucosal microbial antigens. They have important functions in mucosal immune responses in the oral mucosa (33), the lung (34), and against several infectious pathogens, such as *E. Coli*, *H. Pylori*, and *H. influenzae* (35). MAIT cells also play a role in several types of cancers, such as cervical cancer, where DN MAIT cells have been linked to survival benefit (36), as well as in the development of several autoimmune diseases (37, 38).

#### 2.2.3 TCR γδ^+^ DNT cells

TCR γδ^+^ DNT cells, classified by the expression of TCR γδ, play an important regulatory role (39). They account for over 70% of all γδ T cells (40).

Single cell RNA sequencing has improved our understanding and definition of DNT cells into subgroups, based on differential gene expression. Yang et al. used RNA sequencing on murine immune cells to identify five distinct groups of naïve DNT (nDNT) cells. These included nDNT at rest, nDNT helper, nDNT intermediate, nDNT cytotoxic and innate nDNT. There five groups of nDNT cells have different transcription signatures and roles within the immune system, hence this progresses our understanding of how DNT cells are linked to autoimmune diseases (41).

TCR γδ+ DNT cells are activated by antigen presentation by MHC I, MHC II and CD1 molecules (26). They function as potent cytotoxic effector cells through the release of perforin, granzyme B, IFN-γ, tumor necrosis factor (TNF)-α, IL-4, and IL-17, hence help to combat intracellular and extracellular pathogens and malignant cells (42). In addition to their cytotoxic functions, these cells can downregulate the innate and adaptive immune response by secreting IL-10 and transforming growth factor (TGF)-β. They also contribute to tissue repair and wound healing, through the secretion of epithelial cell growth factors. However, this regulatory activity may also suppress anti-tumor activity, hence highlighting the dual role of TCR γδ+ DNT cells in immune regulation (43).

### 2.3 Heterogeneity and plasticity of DNT cells

DNT cells tend to exhibit effector cell phenotypes with a terminal differentiation status and poor proliferation upon anti-CD3 and TCR complex activation (5). Expanded DNT cells show increased Ki67 expression, narrowed TCR Vβ repertoire, and diluted TREC content during clonal proliferation in SLE and acute kidney injury (44, 45). It may be that there is heterogeneity in the DNT cell pool in differentiation states and potential for plasticity, therefore, some cells may be fully differentiated, whilst others may be partially differentiated (46).

DNT cells display diverse cytokine profiles in different disease models. IL-17 is the main pro-inflammatory cytokine secreted by DNT cells in SLE and chronic infection settings. IL-17 production can be promoted by IL-23 and reduced by IL-2 (47, 48). In contrast, DNT cells produce immunosuppressive IL-10 to regulate immune response in other models, such as in non-obese diabetes and allograft rejection (7, 9, 49). Current evidence suggests naïve DNT cells primarily exhibit regulatory functions to maintain self-tolerance, but chronic inflammation disrupts this balance, leading to predominant pro-inflammatory DNT cells (50).

## 3 The role of DNT cells in the development of autoimmune diseases

A summary of the autoimmune conditions discussed in this section and the evidence for the role of DNT cells is presented in **Table 1**.

**Table 1:** The role of DNT cells in the pathogenesis of different autoimmune diseases described in this article.

Disease	Role of DNT cells	Model	Reference
SLE	Produce IL-4, Ig, and anti-DNA antibodies. Conjuncts with CD4+ T cells which induce systemic inflammation. Trigger the secretion of IL-17 stimulated under CD3. Promotes B cells to produce antibodies.	Human patient and mouse model	(51, 52)
SS	The expression of the B-cell activating factor (BAFF) and the hypomethylation of the TNFSF13B gene encoding BAFF increased. Leads to the expansion of these cells in the peripheral blood and their infiltration into the salivary glands, where they produce IL-17.	Human patient	(53–55)
Psoriasis	DNT cells participate in the development of skin inflammation through the expression of IFN-γ and IL-17.	Human patient and mouse models	(56, 57)
Axial spondylarthritis	Express IL-17 and IFN-γ which contribute to skin inflammation.	Mouse models	(58)
ALPS	Has a close relationship with defective Fas-mediated apoptosis Elevate levels of IL-10, IL-18, and soluble FAS-ligand	Human patient and mouse models	(59–65–67)
T1D	Inhibit the development of autoimmune through immunosuppressive ability induced by CD4+ or CD8+T.	mouse models	(4, 68–71)

### 3.1 Rheumatological conditions

#### 3.1.1 Systemic lupus erythematosus

SLE is a systemic autoimmune disease arising from inappropriate immune responses to self-antigens. This leads to the deposition of autoantibodies and immune complexes, as well as lymphocyte infiltration, which affects several organs including the skin, kidneys, and joints (72). The infiltrating lymphocytes in SLE are composed of activated T cells expressing high levels of adhesion molecules (51).

The population of DNT cells in the peripheral blood of SLE patients are significantly expanded compared to healthy controls (52). They are known to induce systemic inflammation synergistically with CD4^+^ T cells, by producing pro-inflammatory cytokines such as IL-4 and IL-17, and anti-DNA IgG autoantibodies (17, 52, 73). DNT cells have been shown in a murine model to produce IL-17 and proliferate rapidly following anti-CD3 stimulation and exposure to self-antigens, and as noted earlier, the PD1^+^ but not PD1^-^ phenotypes of DNT cells are the main source of IL-17 (25). In a murine model, activation of the mTORC1 pathway in DNT cells is associated with higher IL-17A levels, and phosphatidic acid, a lipid metabolite in T cells, can increase mTORC1 downstream signaling and lead to the expansion of IL-17A-producing DNT cells (11). Moreover, an *in vitro* study showed that DNT cells can also promote B cells to produce autoantibodies, contributing to the development of SLE (52).

It has been reported that a large proportion of the expanded DNT cells is likely derived from self-reactive CD8^+^ T cells (17). The conversion from CD8^+^ T cells into DNT cells is due to the downregulation of CD8 expression induced by the inflammatory cytokine environment. This contributes to the pathogenesis of SLE through the loss of CD8-dependent immunosuppressive mechanisms (17).

#### 3.1.2 Sjögren’s syndrome

Sjögren’s syndrome (SS) is a chronic autoimmune disease which leads to chronic inflammation, tissue damage, and impaired secretory function of exocrine glands, hence the hallmark of the disease is dryness of the eyes and mouth (74, 75).

DNT cell expansion has been reported in the peripheral blood of SS patients, as well as in the lymphocytes which infiltrate into the salivary glands of SS patients (53). These DNT cells from patients with SS produce IL-17, which plays an important role in the destruction of glandular tissues (54), and have been found to show no response to corticosteroids *in vitro* (53). Furthermore, DNT cells from patients with SS promote the pathological activation of B cells by increasing the expression of B cell activating factor (BAFF), via epigenetic modulation of the BAFF-encoding gene, *TNFSF13B* (55). As a member of the tumor necrosis factor (TNF) family, BAFF promotes the maturation and activation of B cells, whereas, an excess of BAFF leading to B cell hyperactivity and autoantibody production are known features of SS (76).

#### 3.1.3 Psoriasis and axial spondylarthritis

Psoriasis is a common systemic autoimmune, characterized by dryness, scaling and redness of the skin (77). Its underlying pathogenesis involves immune cell infiltration into the epidermis, inducing skin tissue inflammation and excessive proliferation of keratinocytes (56). In a murine model of psoriasis, DNT cells were found to infiltrate lesional skin and to produce IL-17, promoting skin inflammation (57). DNT cells isolated from the peripheral blood of psoriasis patients show reduced DNA methylation of the *IFNG* gene (which permits greater production of IFN-γ). These DNT cells also express higher levels of PD-1 (56). Together, these findings indicate that DNT cells are involved in the pathogenesis of psoriasis and contribute to skin inflammation via the expression of IFN-γ and IL-17.

Axial spondylarthritis is another chronic autoimmune disease that affects primarily the spine, the sacroiliac joints and tendon-bone attachments (entheses) but shares many genetic features with psoriasis (78). Interestingly, in a murine model of spondyloarthropathy, DNT cells expressing IL-23 receptor were shown to be present in inflamed entheses, and responded to IL-23 by secreting further inflammatory interleukins and chemokines (58). This supports the role of DNT cells in the pathogenesis of axial spondylarthritis.

### 3.2 Autoimmune lymphoproliferative syndrome

Autoimmune lymphoproliferative syndrome (ALPS) is a rare disorder leading to splenomegaly, non-malignant lymphadenopathy cytopenia and autoimmune complications such as glomerulonephritis, hepatitis and vasculitis (59). ALPS is caused by genetic mutations which lead to defective lymphocyte apoptosis, resulting in the abnormal accumulation of lymphocytes. Notably, one of the diagnostic criteria for ALPS is the expansion of DNT cells in peripheral blood and secondary lymphoid organs, which contributes to the disease’s pathogenesis (60, 61).

The classical genetic mutations causing ALPS are found in FAS, FASL and CASP10, causing defective Fas-mediated apoptosis and hence accumulation of lymphocytes (62). Fas and Fas ligand (FasL) interact, leading to the formation of the death-inducing signaling complex, which triggers a cascade of caspase enzymes which facilitate apoptosis (63). Other biochemical changes associated with ALPS are higher peripheral blood levels of IL-10, IL-18, and soluble FAS-ligand (62).

DNT cells from ALPS patients may also be derived from CD8^+^ T cells because the two T cell types share a significant proportion of CDR3 sequences across several TCRVβ families (64). They express abnormally high levels of the B cell antigen B220 (an isoform of the CD45 antigen), which is also expressed on activated T cells that undergo apoptosis (65). DNT cells from ALPS patients co-express CD27 and CD28, which is a characteristic of naïve and central memory T-cell subsets and is usually absent in effector T cells (66, 67). Despite this, DNT cells are mature antigen-experienced effector T lymphocytes, and DNT cells from ALPS patients do proliferate, in contrast to DNT cells from healthy individuals (32, 61).

The role of the accumulation of DNT cells in contributing to the pathogenesis of ALPS is well evidenced, based on several studies. The presence of autoantibodies in most ALPS patients correlates with the abundance of DNT cells in peripheral blood, which may be due to the B cell-promoting effects of IL-10, which is highly expressed by DNT cells from ALPS patients (63, 79). Furthermore, effective treatment of ALPS in murine models (using rapamycin) improved the pathological features of ALPS (such as splenomegaly, lymphadenopathy), in association with decreasing DNT cell levels and autoantibody levels (80, 81). Whilst this supports the key role of DNT cells in the pathogenesis of ALPS, further evidence is still needed to elucidate the pathophysiological mechanisms.

### 3.3 Type 1 diabetes

Type 1 diabetes (T1D) is an autoimmune disease in which there is destruction of insulin-producing pancreatic β cells, caused by self-reactive CD4^+^ and CD8^+^ T cells targeting β -cell antigens in genetically susceptible individuals (82, 83).

DNT cells have been found to prevent the development of autoimmune diabetes through their immunosuppressive effects (9), which is supported by several studies. Firstly, in the non-obese diabetic (NOD) murine model of T1D, diabetes develops around 12–14 weeks of age, which is correlated with a decrease in DNT cell numbers at the same time (49). Next, several studies using different diabetic mouse models show that transfer of DNT cells can slow the progression or even reverse autoimmune diabetes (68–70). Furthermore, transfer of NOD CD8^+^ T cells resulted in diabetes but co-transfer of NOD CD8^+^ T cells with DNT cells did not. It suggests that DNT cells act directly on the pathogenic effector T cells to carry out their immunoregulatory function (49). This may be through Fas/FasL-mediated apoptosis (71), perforin-mediated killing (84, 85) or the modulation of antigen-presenting cells through secretion of IL-10 and IFN γ (68, 86).

## 4 DNT cells in autoimmune disease immunotherapy

### 4.1 Roles and applications of DNT cells in treating autoimmune diseases

**Figure 3:**
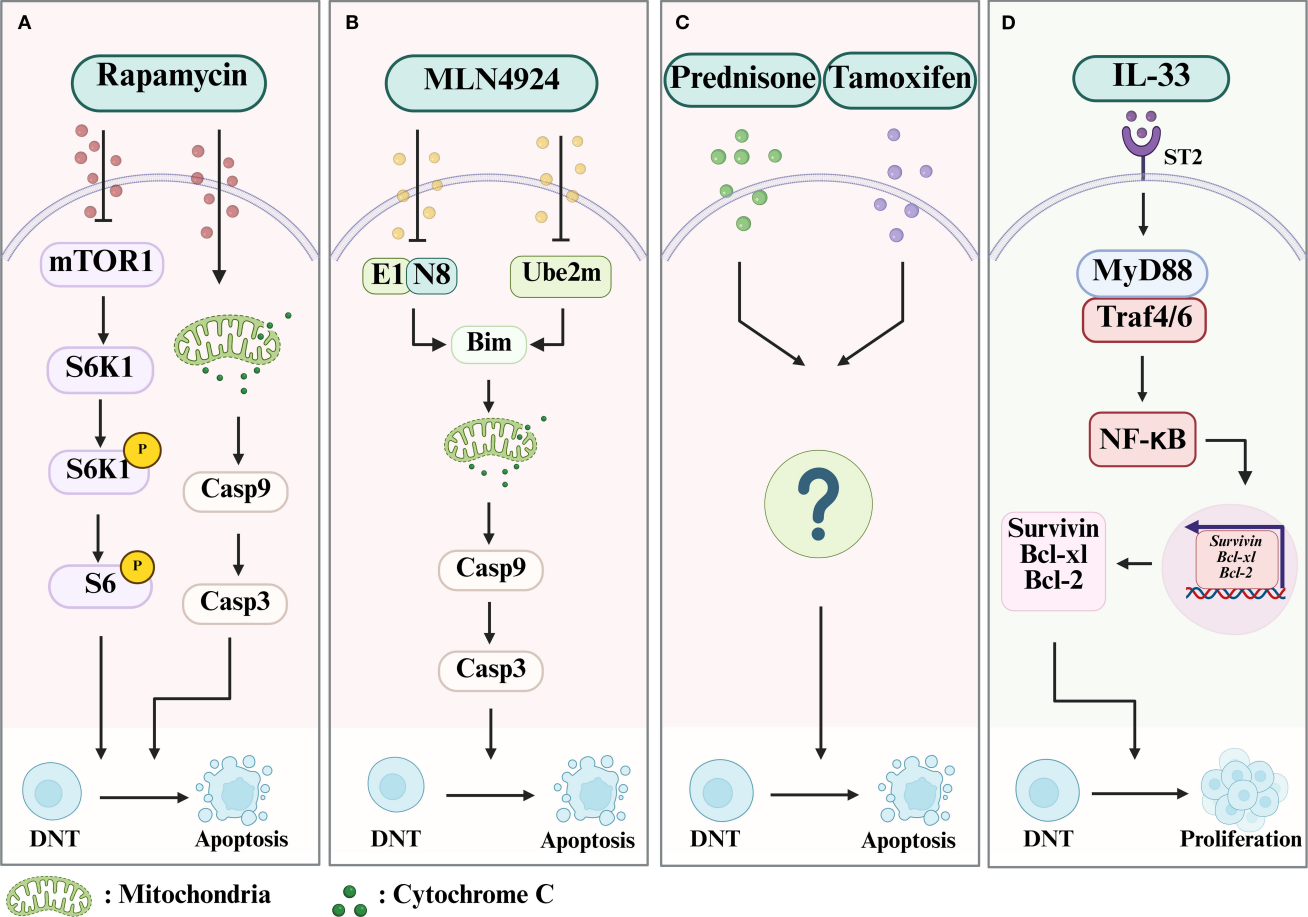
The mechanism of drugs which limit or promote the expansion of DNT cells. **(A)** Rapamycin can lead to the apoptosis of DNT cells by both inhibiting the mTOR/S6K1 pathway and Cytochrome C/Casp 9/Casp 3 pathway. **(B)** MLN4924 can inhibit NAE1 or Ube2m to block degradation of Bim, which will increase the mitochondrial membrane permeability and lead to the activation of the Cytochrome C/Casp 9/Casp 3 pathway, resulting in the apoptosis of DNT cells. **(C)** Prednisone and Tamoxifen can promote the apoptosis of DNT cells via an undefined mechanism. **(D)** IL-33 can contribute to the survival and proliferation of DNT cells by activating the MyD88/NF-κB pathway, which will subsequently induce the expression of Bcl-xl, Bcl-2, and Survivin, through the ST2 receptor. Case, Caspase; NEDD8, neuronal precursor cell-expressed developmentally downregulated protein 8; NAE1, NEDD8-activating enzyme E1; Ube2m, genetic knockout of NEDD8-conjugating enzyme E2.

As discussed, DNT cells display diverse functions (including both inflammatory and anti-inflammatory activity) in autoimmune diseases. Therefore, there are several potential therapeutic approaches to autoimmune diseases which use our understanding of the role of DNT cells, such as selective inhibition to render DNT cells less pathogenic, specific modulation to promote their proliferation and inhibiting their apoptosis. Increasingly, approaches directly or indirectly targeting DNT cells have been trialed. Multiple potential approaches are depicted in **Figure 3**.

#### 4.1.1 Drug treatments limiting the expansion of pathogenic DNT cells

Rapamycin (also called sirolimus) is a drug which inhibits the mammalian target of rapamycin (mTOR) and is an immunosuppressive and antitumor agent with a tolerable side-effect profile (87). which may induce apoptosis in T and B lymphocytes (88, 89). In a preclinical study with murine models of ALPS, rapamycin induced a dramatic decrease in DNT cells (both in peripheral blood and lymphoid tissue), as well as clinical signs of the condition and the levels of anti-dsDNA IgG autoantibodies) (80). The authors also found that rapamycin could downregulate phosphor-S6 by inhibiting the activation of S6K1 (p70S6 kinase) and phospho-Bad (a proapoptotic molecule that is regulated by phosphorylation), and hence trigger caspase-dependent apoptosis. Subsequently, the function of rapamycin in suppressing the proliferation of DNT cells was verified again in a clinical trial including six ALPS patients who had failed therapy with steroids, where all patients showed significant response to treatment. It was suggested that rapamycin could be an effective second-line treatment for ALPS (81). A similar result was reported in a patient with a *de novo* FAS mutation with a severe phenotype of ALPS (90). Peripheral blood samples from the case report patient showed T-cell lymphocytosis with a higher proportion of DNT cells and a lower proportion of Treg cells compared to healthy controls. The patient displayed rapid clinical improvement and decrease in DNT cell numbers after being started on rapamycin.

Resembled to ubiquitination, neddylation-a form of protein post-translational modification involving binding of neuronal precursor cell-expressed developmentally downregulated protein 8 (NEDD8) to other proteins-is known to regulate T cell immune responses. It plays an important role in regulating CD4+ T cell activation, proliferation, and differentiation into T helper subsets such as Th1, Th2, T follicular helper cells and Treg cells (91–93). In a murine lupus model using MRL/l*pr* mice, it has been shown that inactivation of neddylation with MLN4924, a specific inhibitor of NEDD8-activating enzyme E1 (NAE1), or genetic knockout of NEDD8-conjugating enzyme E2 (Ube2m) in T cells, decreased DNT-cell accumulation and slowed the development of lupus (94). Further investigations revealed that inactivation of neddylation reduced the degradation of Bim (Bcl-2 interacting mediator of cell death, a pro-apoptotic protein) by ubiquitination and maintained Bim at higher levels in DNT cells, hence contributing to the apoptosis of the expanded DNT cells in lupus mice. In double knockout lupus mice (*Ube2m* ^-/-^ *Bim* ^-/-^ *lpr*), the inactivation of Bim prevented increase in apoptosis of DNT cells and the anti-lupus effect seen when expression of *Ube2m* alone was inhibited, suggesting that the apoptosis triggered by neddylation is dependent on Bim (94). Compared to healthy controls, SLE patients are known to have lower levels of Bim and higher levels of Cullin1 neddylation, and the inhibition of neddylation can cause apoptosis of SLE patient DNT cells (94). Altogether, these new findings suggest a promising therapeutic approach to SLE through targeting DNT cell neddylation.

Glucocorticoids are effective in treating inflammation in many autoimmune conditions, including in the induction and maintenance of remission in patients with SLE (95). CD138 (syndecan-1) is a member of the transmembrane heparan sulfate proteoglycan family and is important in mediating cell-cell adhesion and cell-matrix adhesion (96). It is also a marker of differentiated plasma cells and is associated with the production of anti-dsDNA antibodies in the pathogenesis of SLE (97). A large proportion of DNT cells in MRL/*lpr* mice (a model of spontaneous SLE) are CD138 positive (CD3^+^CD4^-^CD8^-^CD138^+^ phenotype) (98). Similar to the pathogenesis of ALPS, it has been reported that Fas deficiency leads to DNT cell accumulation in the spleens of MRL/*lpr* mice (a model of spontaneous SLE) and results in splenomegaly and lymphadenopathy (99). The CD138^+^ DNT cells which accumulate in the spleens of MRL/*lpr* mice overexpress FasL, and therefore can attack tissues in MRL/*lpr* mice that express low levels of the Fas receptor (100). Prednisone, a commonly used GC, when used to treat SLE in MRL/*lpr* mice, cause reduced accumulation of CD138^+^ T cells in the spleens of MRL/*Lpr* mice. This also leads to prednisolone causing reduced levels of anti-dsDNA autoantibodies (101). This finding provides new insights into the therapeutic effects and mechanisms of GCs as treatment for SLE, although the signaling pathways for reducing the frequency of CD138^+^ DNT cells in MRL/*lpr* mice are yet to be elucidated.

As a selective estrogen receptor modulator, tamoxifen (TAM) is used to treat estrogen receptor-positive breast cancer. Its therapeutic effect is mediated by endoxifen, the principal metabolite of tamoxifen (102). It is a synthetic non-steroidal triphenylethylene compound (103). Studies using MRL-*lpr/lpr* murine SLE models have shown that estrogen treatment can exacerbate the pathologies in SLE, whilst TAM induces positive response to treatment, such as reducing splenomegaly and lymphadenopathy (104–107). In addition, TAM-treated MRL-*lpr/lpr* mice had a significantly lower proportion of DN T cells (108). *In vitro*, TAM also led to dose-dependent inhibition of the IL-2-activated proliferation of DNT cells from lymph nodes (108). These findings offer a novel approach to exploring the feasibility of using selective estrogen receptor modulators as treatment for autoimmune diseases (including SLE).

#### 4.1.2 Specific drugs to promote the proliferation and activation of DNT cells

Autoimmune hepatitis (AIH) is an inflammatory disease of the liver mediated by abnormal autoimmune responses, which can occur in different age groups and ethnic groups, and is more common in women (109). In 2021, the global incidence of AIH was 0.4-2.39/100,000 people, and the global prevalence was 4.8-42.9/100,000 people, and the incidence and prevalence continue to increase yearly (110). It is widely understood that liver injury is induced by the aberrant activation of T cells (mainly Th1 and Th 17), B cells, and macrophages directed against liver autoantigens, due to a loss of tolerance (111).

A recent study demonstrated that transfer of DN T cells converted from CD4^+^ T cells could suppress inflammatory responses in hepatitis, using a typical Concanavalin (Con A)-induced murine liver injury model (112). IL-33 is a cytokine released during tissue injury, with important roles in regulating inflammation, tissue homeostasis and repair and type 2 immune responses, as well as affecting the development of autoimmune diseases, viral infections, and cancer (113). It activates many immune cell subsets including NK cells, CD8^+^ T cells, neutrophils, regulatory macrophages, and Treg cells, via the IL-33 (ST2) receptor (114). Recent studies have found that DN T cells also express ST2 and that stimulation by IL-33 promotes DN T cell proliferation and suppresses their apoptosis, both in the Con A-induced murine liver injury model and *in vitro* during the conversion of DNT cells from CD4^+^ T cells isolated from human peripheral blood (112). In conclusion, these studies reveal a role for IL-33 in regulating DN T cells, and introduce new potential pathways to induce the expansion of DNT cells in the immune environment.

### 4.2 Combination of Chinese medicinal materials and DNT cells in treating autoimmune diseases

It has been shown that various traditional Chinese medicines (TCMs) can ameliorate autoimmune diseases, including in rheumatoid arthritis, osteoarthritis SLE and AIH, which has been demonstrated by both clinical trials and preliminary research *in vivo* (115–117). These TCMs include monomers isolated from Chinese herbs, extracts of Chinese herbs, and Chinese medicinal formulae which have been applied for centuries. TCM treatment focuses on regulating the body’s immune microenvironment and has unique advantages in the treatment of autoimmune diseases. These advantages are mainly reflected in the following aspects: it can adapt to the functional complexity of DNT cells, reduce disease recurrence, is suitable for elderly and frail patients, improve the universality of treatment, and alleviate the side effect of immune hypofunction caused by modern medicines (118). There is potential to integrate TCM and Western medicine in the approach to treating autoimmune diseases.

**Figure 4:**
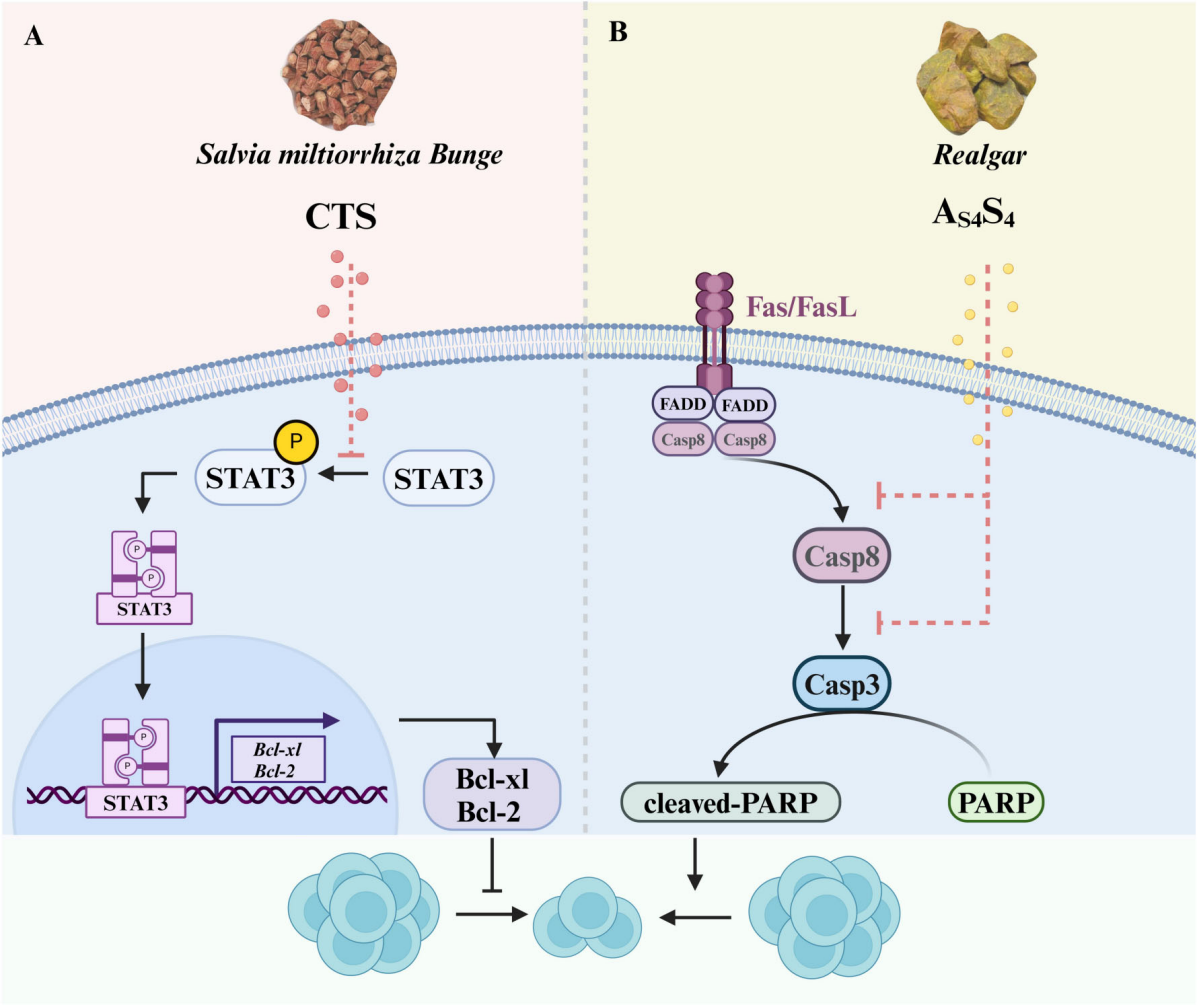
Effects of CTS and As4S4 on the differentiation of DNT cells. **(A)** CTS, which is isolated from *Salvia miltiorrhiza Bunge* (Danshen), can inhibit the proliferation of DNT cells by blocking the phosphorylation of STAT3 to reduce the expression of Bcl-xL and Bcl-2. **(B)** As_4_S_4_, derived from *Realgar*, can trigger Fas-mediated apoptosis of DNT cells by activating the Caspase8/Caspase 3 pathway. CTS, Cryptotanshinone; As_4_S_4_, Tetra-arsenic Tetra-sulfide.

There are thus far few studies investigating the use of TCM treatments to regulate DNT cells in autoimmune disease. Of note, the effect of zuoguiwan (a typical TCM documented in the Complete Compendium of Zhang Jingyue) on thymic differentiation of DNT cells has been studied (119). In the embryonic rat thymus, zuoguiwan induces T-cell differentiation in the thymus from the DN4 stage to the DP stage and inhibits the development of DNT cells by increasing the secretion of thymosin *β4* and thymosin α1 (119). Huanglian Jiedu Decoction (HLJD) can influence the changes of DNT cells in the treatment of ischemic stroke. Specifically, it can regulate activated cytotoxic DNT cells and quiescent immunosuppressive DNT cells (120). In the treatment of SLE, the Jieduquyuziyin prescription (JP) therapy can induce the apoptosis of DNT cells by inhibiting the neddylation pathway to restore their homeostasis, while Ube2m serves as a crucial therapeutic target for the JP therapy in regulating the homeostasis of DNT cells (121). Several other active ingredients extracted from TCM herbs have been shown to treat autoimmune diseases by effectively modulating DNT cell differentiation (122, 123). They are summarized in **Figure 4**.

Cryptotanshinone (CTS), a key bioactive quinoid diterpene derived from the dried roots of Salvia miltiorrhiza Bunge (Danshen, a traditional Chinese herbal medicine), has been utilized in the management of cardiovascular, cerebrovascular, and autoimmune disorders (122, 123). It is an effective inhibitor of STAT3 (124), which is overactive in the development of SLE (125).

STAT3 is activated by kinase-mediated phosphorylation at Tyr705, and two phosphorylated STAT3 dimerize to form a transcription factor complex that translocates to the nucleus to induce target gene expression (126). In a preclinical study using CTS to treat SLE in MRL/*lpr* mice, CTS decreased the total number of T cells and especially DNT cells. CTS treatment also led to a decrease in levels of autoantibodies and pro-inflammatory cytokines. Clinically, CTS treatment improved the signs and symptoms of SLE, such as reducing the size of skin lesions, reversing splenomegaly and improving kidney function (122). This study also showed that CTS reversed the excessive STAT3 signaling in the spleens of lupus-prone MRL/*lpr* mice, and that inhibition of T cell proliferation was through the inhibition of STAT3 activation (122). This suggests that CTS, as a potential therapeutic drug for SLE patients, can block DNT cell proliferation through reducing STAT3 phosphorylation and hence activation, to attenuate the development and progression of SLE.

Tetra-arsenic tetra-sulfide (As_4_S_4_), the major active ingredient of the traditional Chinese medicine realgar, is known to have little toxicity and has long been used for treating various diseases, including SLE and leukemia (127, 128). As_4_S_4_ treatment of lupus-prone BXSB mice reversed monocytosis in the spleen and decreased serum IL-6 levels (127). Furthermore, As_4_S_4_ in lupus-prone MRL/*lpr* mice significantly decreases the number of DNT cells, reduces their production of IL-17 and the production of antinuclear antibodies (129). The As_4_S_4_-induced decrease in DNT-cell number could be due to increased apoptosis, as suggested by the findings of decreased FasL expression and activation of several caspases after treatment (129). This study strongly supports the therapeutic potential of As_4_S_4_ in SLE through the suppression of DNT cells.

**Table 2** provides a summary of the discussed therapeutic approaches using DNT immunotherapy to treat autoimmune diseases.

**Table 2:** DNT cell immunotherapy in preclinical and clinical applications in autoimmune diseases.

Drugs or approach	Effect on the expansion or proliferation	Targets	Preclinical or clinical	Reference
Rapamycin	Reduce the amplification of DNT cells	mTOR	Clinical and Preclinical	(80, 81, 87–89)
Neddylation inhibitor MLN4924	Reduce the accumulation of DNT cells	NEDD8-activating enzyme E1 or genetic abrogation of NEDD8-conjugating enzyme E2 (Ube2m)	Clinical and Preclinical	(91–94)
GCs	Reduce the amplification of DNT cells	Unknown	Clinical	(95–101)
Tamoxifen	Reduce the proportion of DNT cells	Estrogen receptor	Clinical	(102–108)
IL-33	Promote the proliferation and survival of DNT cells	ST2 receptor	Preclinical	(112)
zuoguiwan	induces T-cell differentiation in the thymus from the DN4 stage to the DP stage and inhibits the development of DNT cells by increasing the secretion of thymosin β4 and thymosin α1	thymosin β4 and thymosin α1	Preclinical	(119)
Huanglian Jiedu Decoction	regulate activated cytotoxic DNT cells and quiescent immunosuppressive DNT cells	Unknown	Preclinical	(120)
Jieduquyuziyin prescription	induce the apoptosis of DNT cells by inhibiting the neddylation pathway to restore their homeostasis	Neddylation pathway	Preclinical	(121)
Cryptotanshinone	Block the proliferation of DNT cells	STAT3	Preclinical	(122)
Tetra-arsenic tetra-sulfide	Reduce the number of DNT cells	FasL/Caspase pathway	Preclinical	(127)

## 5 Potential limitations and challenges

Despite the relatively small numbers of DNT cells in peripheral blood and lymphoid organs, their immunoregulatory and pathogenic activities in the development and progression of autoimmune diseases have been widely acknowledged. Targeting the differentiation and expansion of DNT cells has potential as a valuable new approach for treating autoimmune diseases. The discovery of potential targets, including the IL-33 receptor, ST2 (112), mTOR (81), and STAT3 (122), for regulating DNT cells lays the foundation for the development of targeted drugs. Nonetheless, there are still some limitations and challenges in the application of DNT cells in treating autoimmune diseases.

Firstly, DNT cells perform diverse functions in different autoimmune diseases, and hence this may mean that different strategies to regulate DNT cells are required depending on the disease. The complexity of studying the role of DNT cells in different autoimmune disease settings is due to their phenotypic and functional diversity in different immune environments and tissues. DNT cells can inhibit the progression of autoimmune diabetes through their immunosuppressive functions, such as IL-10 secretion (4, 9, 68). On the other hand, DNT cells proliferate and produce IL-17 in SLE, leading to increased inflammation and tissue damage (17, 25). Therefore, the choice of whether to promote or inhibit DNT-cell activity requires consideration on a case-by-case basis for different autoimmune conditions. It will be crucial to elucidate the gaps in knowledge about the roles of DNT cells and their interactions with other immune cells in different pathologies and tissue types.

Secondly, most of the molecular targets that regulate the differentiation and expansion of DNT cells have multiple biological functions, and direct intervention may lead to unexpected side effects. For example, although mTOR could be a potential target to regulate the differentiation of DNT cells, mTOR activation also inhibits the functions of CD8^+^ effector memory T cells and CD4^+^ Treg cells, which may lead to disruption of immune homeostasis (130).

Additionally, the controversy on the origin of DNT cells continues. There are several hypotheses summarized in **Figure 1**. The lack of clear understanding of the ontogeny of DNT cells makes it difficult to regulate DNT cells from their origin, hence current approaches can only target the DNT cells which are already differentiated.

Another consideration is that several studies have suggested that adoptive DNT cell therapy may serve as a strategy to treat autoimmune diseases such as T1D (47, 71). However, the survival time of DNT cells *in vivo* limits its application. The human DNT cells can persist *in vivo* for several weeks (131), then, reinfusion of cryopreserved DNT cells may be required once DNT cell levels decrease below an established threshold. This means that the interval and number of cycles for DNT cell infusion need to be investigated before a treatment protocol using adoptive DNT cell therapy can be established. We can use gene editing technologies to enhance the functional activity of DNT cells, or developing strategies for long-term cell-based therapeutic regimens.

Finally, most studies investigating DNT cells are based on models using immunodeficient mice, hence, further clinical trials with larger study populations would need to be carried out to assess the efficacy of potential treatments in humans.

## 6 Conclusion

As a rare but important subset of mature T lymphocytes, DNT cells play a vital role in immune homeostasis. They can function as Treg cells, cytotoxic T cells, or Th cells and regulate the innate and adaptive immune systems. They are also closely linked with the development of several autoimmune diseases. A growing understanding of DNT cell origin and functional features has prompted the consideration of DNT-cell-based therapeutic approaches, including precise modulation of DNT cell activation and survival and inhibition of proinflammatory metabolic pathways. In brief, by summarizing the current understanding of the role of DNT cells in autoimmune disease, we have highlighted the heterogeneity and diverse functions of DNT cells. Hence, there is still a need for the use of DNT cells as potential therapeutic tools to be further explored, in the era of precision and personalized medicine.
